# A microfluidic approach for label-free identification of small-sized microplastics in seawater

**DOI:** 10.1038/s41598-023-37900-9

**Published:** 2023-07-07

**Authors:** Liyuan Gong, Omar Martinez, Pedro Mesquita, Kayla Kurtz, Yang Xu, Yang Lin

**Affiliations:** 1 Department of Mechanical, Industrial and Systems Engineering, University of Rhode Island, Kingston, RI USA; 2 Department of Civil and Environmental Engineering, University of Rhode Island, Kingston, RI USA; 3 Department of Computer Science, San Diego State University, San Diego, CA USA

**Keywords:** Mechanical engineering, Marine chemistry, Environmental sciences

## Abstract

Marine microplastics are emerging as a growing environmental concern due to their potential harm to marine biota. The substantial variations in their physical and chemical properties pose a significant challenge when it comes to sampling and characterizing small-sized microplastics. In this study, we introduce a novel microfluidic approach that simplifies the trapping and identification process of microplastics in surface seawater, eliminating the need for labeling. We examine various models, including support vector machine, random forest, convolutional neural network (CNN), and residual neural network (ResNet34), to assess their performance in identifying 11 common plastics. Our findings reveal that the CNN method outperforms the other models, achieving an impressive accuracy of 93% and a mean area under the curve of 98 ± 0.02%. Furthermore, we demonstrate that miniaturized devices can effectively trap and identify microplastics smaller than 50 µm. Overall, this proposed approach facilitates efficient sampling and identification of small-sized microplastics, potentially contributing to crucial long-term monitoring and treatment efforts.

## Introduction

Microplastic pollution has become a global concern, and it is estimated that there are approximately 24.4 trillion pieces of microplastics in the upper ocean, emphasizing the extensive presence of this pollutant in marine environments^1^. Over time, the cumulative impact of microplastic pollution on marine biota has resulted in significant health threats, posing a serious risk to the entire ecosystem^2^. Efficient sampling, accurate identification, and reliable chemical characterization of microplastics are crucial to understanding their environmental and biological impacts. Nevertheless, the lack of systematic processes persists due to the intricate nature of environmental microplastics, encompassing factors such as their varying sizes, shapes, degradation stages, aggregation, and the presence of associated biofilms. Currently, there are three major areas of focus when it comes to studying marine microplastics: sampling, sample treatments with contamination control, and microplastic identification^3^. Ideal sampling enables a high-fidelity collection of microplastics that retains all necessary information acquired naturally without unwanted cross-contamination. However, conventional sampling and separation methods, such as density separation, visual separation, and passive floating, are limited in their ability to effectively separate small particles at the submicron scale^4^, which in fact account for the majority of microplastics in seas. Other methods, such as acidic digestion and enzymatic digestion, are costly processes and may involve the use of highly toxic chemicals that could potentially damage the integrity of the samples^5^. Another area of concern is the potential for cross-contamination from sampling devices and atmospheric particles, which can introduce additional challenges in accurately assessing and quantifying microplastic pollution^6^. Though mitigation strategies such as measuring blank samples can help minimize experimental errors, these methods only eliminate contaminations in the central laboratory^7^. As emphasized in a review from Hidalgo-Ruz et al.^8^ who summarized traditional methodologies in 68 studies of marine microplastics, developing effective methodologies that distinguish more size fractions, prevent contamination, and allow for effective identification and characterization are still a critical task in the field.

Microfluidic technology has been proven to be a powerful tool for particle sorting and separation nowadays thanks to its advantages such as cost saving, rapid response, high throughput, and adaptability in many applications^9,10^. Recent studies revealed that its capabilities have been extended to microplastics research^11–15^. For instance, Elsayed et al.^16^ reported a micro-optofluidic analysis platform to sort microplastic particles in tap water. The sorted microplastics (1–100 µm) were trapped in micro-filters for both Raman and Fourier-transform infrared spectroscopy (FTIR) chemical characterization. However, undesirable accumulation of particles resulted in mixed Raman peaks that unnecessarily increased the difficulties of sample characterization.

Lastly, accurate identification is another essential step in marine microplastic characterization. Currently, the two most common chemical identification techniques are Raman spectroscopy and FTIR. The latter one is a reliable method for analyzing microplastics, yet requirements of dry and relatively large samples (> 10 µm) have limited its applications^17,18^ On the other hand, Raman spectroscopy presents several advantages, including higher resolution and easy sample preparation, enabling the identification of particles with sizes near 1 µm. More importantly, this method is also applicable to liquid samples, even at the microscale^19,20^.

Matching the spectra (both Raman and FTIR) with reference spectra is now widely regarded as the gold standard method for microplastic identification. However, the accuracy and efficiency of this method are hindered by several factors. The accuracy of the mathematical spectrum matching scheme is significantly influenced by the quality of the signal^21^, and unfortunately, this quality can be compromised by fluorescence and luminescence effects. The absence of a comprehensive reference database tailored to different environmental samples poses a challenge for sample identification^22^, and the process itself is often labor-intensive and reliant on expert judgments.

Thanks to the rapid developments of machine learning technologies, models can improve their performance based on customized datasets and make appropriate judgments without human assistance when new samples are discovered. These technologies not only enable powerful feature extraction and classification, but also exhibit high accuracy, flexibility, and adaptability^23^. To the best knowledge of the authors, only one study has been reported in Raman-based microplastic identification using machine learning technologies^24^, yet more accurate identification approaches applicable to more plastic types with size down to microscale are still in demand.

**Figure 1:**
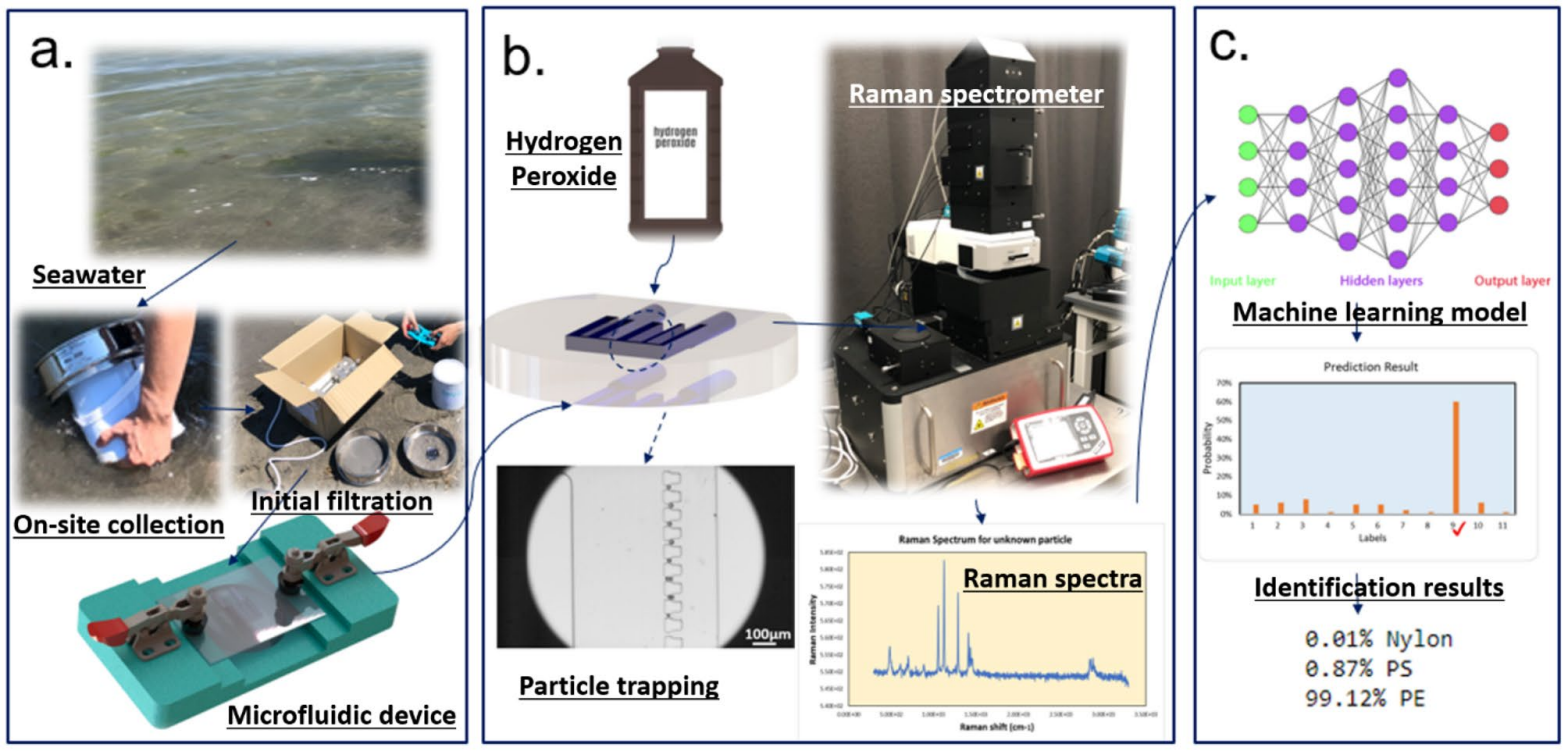
Schematic of the streamlined marine microplastic identification process. (**a**) An assembled microfluidic device is utilized for on-site collection of samples from seawater, which undergoes initial filtration using a 45 µm filter, (**b**) After safely transporting the device back to the laboratory, a solution of 30% (v/v) hydrogen peroxide is injected into the device, where it is left for 16 h to facilitate sample processing. Subsequently, Raman spectra are acquired from the processed samples; (**c**) Machine learning identification process incorporated in the microfluidic-based method for marine microplastic analysis.

In this study, we propose a novel approach for streamlining the process of sampling and identifying marine microplastics. Our method integrates the benefits of microfluidics, Raman spectroscopy, and machine learning technologies, with the aim of improving the efficiency and accuracy of microplastic analysis in marine environments (Fig. 1). Specifically, we constructed a comprehensive training dataset by combining samples collected in the laboratory with publicly available datasets. Our study involved a systematic investigation to evaluate and compare the identification performance of four machine learning models: the support vector machine (SVM) model, random forest (RF) model, convolutional neural networks (CNN), and ResNet34 architectures. Our findings reveal that the CNN model outperforms the other models, with an average classification accuracy of 93%. To demonstrate the efficacy of microfluidic devices in trapping and identifying microplastics, we introduce a poly(dimethylsiloxane) (PDMS) device featuring sieve-like structures designed specifically for capturing small-sized particles. In a proof-of-concept experiment, all trapped pristine particles were accurately identified. Additionally, we successfully validate the practicality of field sampling and identification by utilizing samples collected from a local beach (Narragansett, RI, USA).

## Results and discussion

### Classification performance of machine learning models

**Figure 2:**
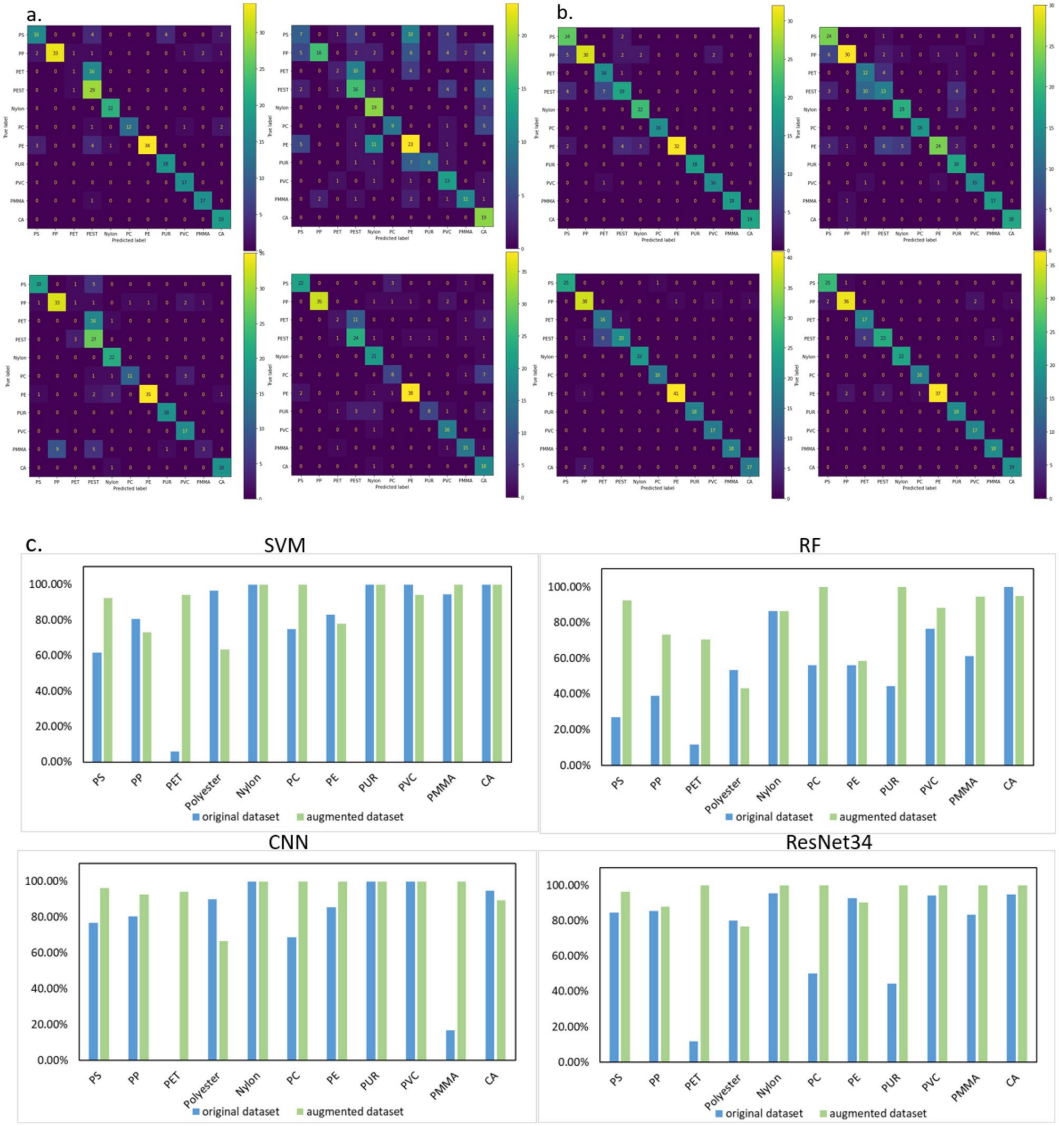
Confusion matrices depicting the classification accuracy of four machine learning models on the test dataset. (**a**) Models were trained with the original training dataset; (**b**) Models were trained with the augmented training dataset. (**c**) Comparisons of identification accuracy on the test dataset for each plastic type based on the original and augmented training datasets.

We first trained the models with the original training dataset and evaluated the classification performance on the test dataset. The confusion matrices are shown in Fig. 2a and the evaluation metrics of accuracy, F1, and MCC scores are summarized in Table 1**.** The best average classification accuracy of 82.0% was achieved by the SVM model, outperforming both the CNN and ResNet34 models, which only reached 77% accuracy. These findings contradict previous studies that suggested superior accuracy for CNN and ResNet models, indicating that the original training dataset was insufficient for effectively training deep learning models^25,26^. To address this issue, we retrained the models using an augmented dataset and reevaluated their classification performance on the testing dataset. Figure 2b illustrates the confusion matrices derived from the augmented training dataset, while Table 2 presents a comparison of evaluation metrics Notably, all models exhibited significant improvements in accuracy results. Figure 2c shows the enhancements in classification accuracy for each class when comparing the original training dataset to the augmented training dataset. Specifically, the accuracy of the SVM, RF, CNN, and ResNet34 models increased by 4.8%, 23.7%, 16.5%, and 15.4%, respectively. The comparable performance of CNN and ResNet34 on both training datasets can be attributed to the shared convolutional feature extraction layers in these two architectures. However, the sensitivity of different models to varying data sizes highlights the need for a more detailed analysis of the relationship between the models and the training datasets^27^. Merely increasing the number of training data points is insufficient for effectively comparing the advantages of these two models. However, when incorporating additional material types and environmental samples with complex degradation conditions, the performance disparity between these two models becomes more discernible. With the implementation of data augmentation, both the CNN and ResNet34 models demonstrated high accuracy, surpassing 93%. In comparison, the SVM model achieved an accuracy of 86.8%. These findings highlight the superior performance of CNN and ResNet34 models, further corroborating previous studies that have reported their efficacy in handling larger datasets^28^. Nevertheless, it is worth mentioning that the low accuracy obtained by the RF model contradicts the results of Ramanna et al. 2022^29^. In light of this, the RF model will not be utilized in this study. Given the comparable performances of CNN and ResNet34, we have opted to proceed with CNN for further demonstrations in this paper.

**Table 1:** Model performance based on the original training dataset (587 data points).

Evaluation metrics	SVM	RF	CNN	ResNet34
Accuracy	82.0%	54.0%	76.7%	77.8%
Macro average F1 score	0.806	0.539	0.743	0.766
Weighted average F1 score	0.793	0.530	0.716	0.722
MCC score	0.806	0.495	0.746	0.756

**Table 2:** Model performance based on the augmented training dataset (11,772 data points).

Evaluation metrics	SVM	RF	CNN	ResNet34
Accuracy	86.8%	77.7%	93.2%	93.2%
Macro average F1 score	0.887	0.799	0.939	0.934
Weighted average F1 score	0.869	0.777	0.932	0.932
MCC score	0.856	0.757	0.925	0.925

To elucidate the classification accuracy for all plastic types, the improvements of each material type are compared side by side (Fig. 2c**).** The classification accuracy for materials such as nylon, polyvinyl chloride (PVC), and cellulose acetate (CA) remains high for all models trained with both training datasets. The identification of the three most abundant plastic types, polystyrene (PS), polypropylene (PP), and polyethylene (PE) was improved after data augmentation, with PE being improved most significantly. Moreover, the classification between Polyethylene terephthalate (PET) and polyester improved substantially after training with the augmented dataset given the fact that both materials have similar chemical compositions. This result highlights the potential for precise identification by introducing additional materials with similar chemical compounds but slightly different physical structures, such as high-density polyethylene (HDPE), low-density polyethylene (LDPE), nylon 6, nylon 6, 6, and many others. When dealing with environmental samples, it is inevitable to encounter variations in spectra due to complex weathering conditions and the presence of diverse additives in the products. These factors contribute to disparities in the spectral data, making precise identification more challenging. It is worth noting that in the case of severely weathered microplastics, certain Raman fingerprint bonds may be lost due to extensive photooxidation, physicochemical changes, and microbial degradation^31^. In such scenarios, relying solely on Raman spectroscopy analysis for accurate identification may not be sufficient. The improvements of machine learning models through the integration of more comprehensive datasets from scanning electron microscopy-energy dispersive X-ray spectroscopy (SEM–EDS) can be considered as an additional approach for element composition identification and surface morphology analysis.

**Figure 3:**
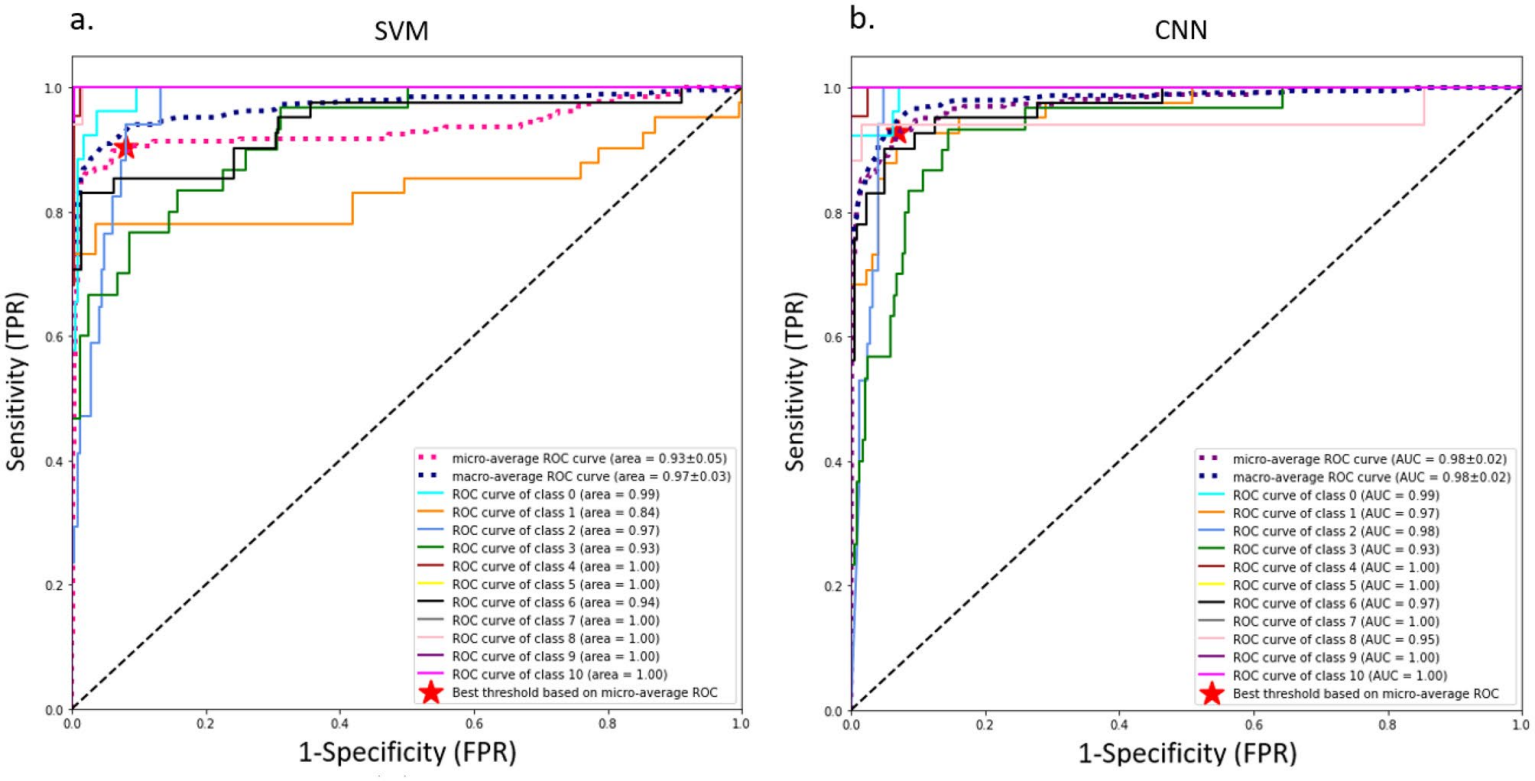
ROC curves and the average AUC evaluation of both SVM and CNN models for all classes based on the OvR method. (**a**) SVM model trained with the augmented training dataset; (**b**) CNN model trained with the augmented training dataset. The classes correspond to different plastic types: 0-PS, 1-PP, 2-PET, 3-Polyester, 4-Nylon, 5-PC, 6-PE, 7-PUR, 8-PVC, 9-PMMA, and 10-CA. Optimal threshold points are calculated based on micro-average ROC curves indicated with the red star.

We further generated the receiver operating characteristic curve (ROC) by plotting the sensitivity against the false positive rate and calculated the average area under the ROC curve (AUC) scores for the SVM and the CNN models using the one versus rest (OvR) method to evaluate the sensitivity and specificity of these two models at various thresholds^32^. As shown in Fig. 3, CNN outperforms SVM, as evidenced by a higher average AUC score of 98 ± 0.02%. In this classification scenario, our aim is to minimize misclassifications by achieving the lowest false positive rate (FPR) and the highest true positive rate (TPR) so that we could maximize the probability to detect positive classes. The false positive rate (FPR) represents the proportion of negative instances or samples that are incorrectly classified as positive. It quantifies the rate at which the model erroneously classifies particles that are not microplastics as one of the trained polymers. At the calculated optimal threshold, the FPR for SVM and CNN models are 0.08 and 0.048 and the TPR are 0.900 and 0.930 respectively. Consequently, the CNN model demonstrates a slightly improved classification performance compared to the SVM model.

### Identification of pristine microplastics trapped in the microfluidic device

**Figure 4:**
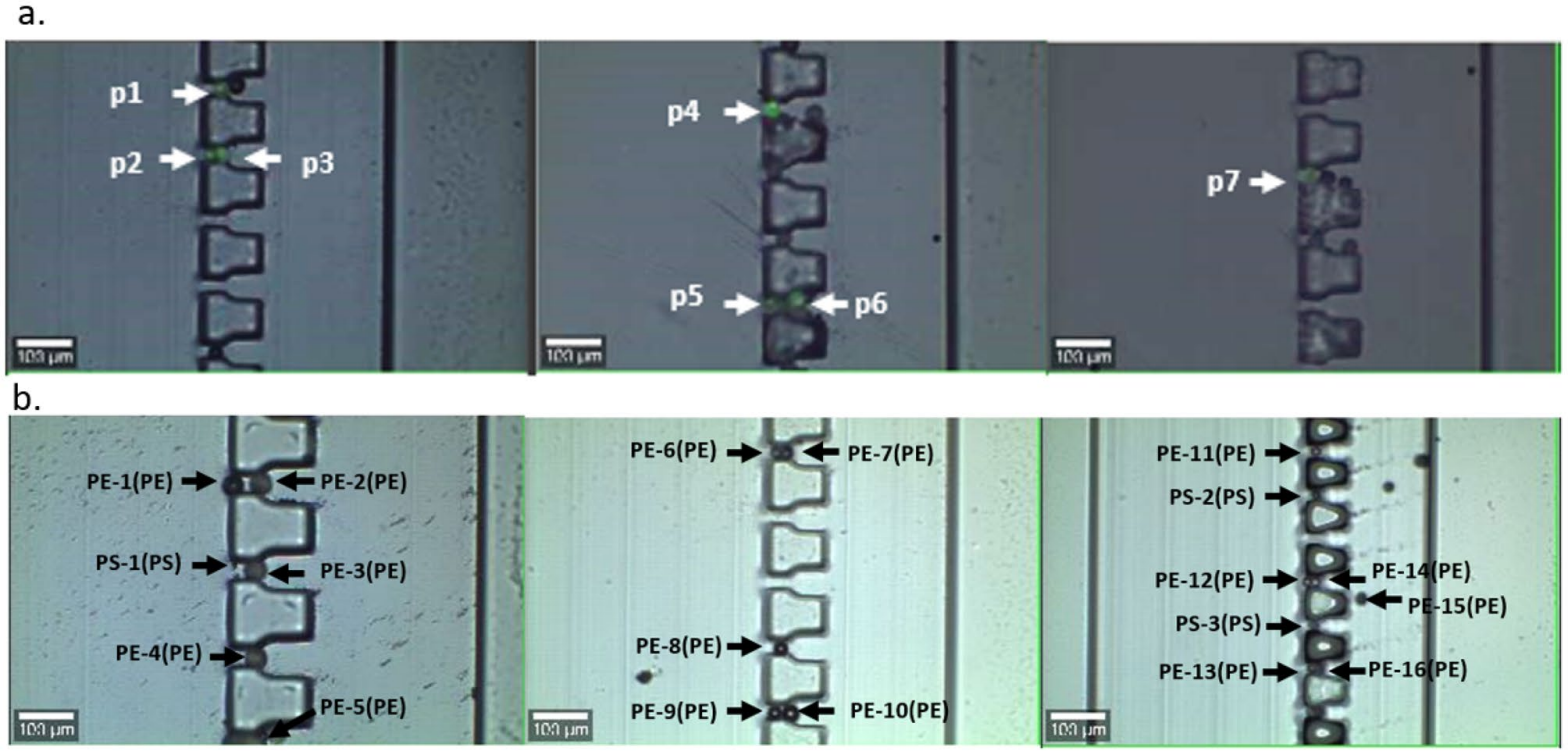
Trapping fluorescent PE particles and regular PE and PS particles in a microfluidic device. (**a**) All 7 selected fluorescence PE particles are trapped in zone B; (**b**) Distribution of the 19 randomly selected particles including 16 PE particles and 3 PS particles. Predicted results are shown in parenthesis.

Besides the performance evaluated on the test dataset, SVM and CNN models were also used to identify small-sized particles trapped in a microfluidic device. Although the models were trained using relatively larger plastics under static conditions along with online databases, the yielded results clearly suggested that accurate identification can be achieved for trapped pristine microplastics, with an accuracy approaching 100%. Specifically, fluorescent PE particles (20-27 µm) were mixed with regular PE (10–45 µm) and PS (9.5–11.5 µm) particles, providing the ground truths for the identification based on fluorescence and size. The results showed that all the fluorescence PE particles were trapped in zone B where the trapping size was designed to be 11-20 µm (Fig. 4a). The Raman spectra of these particles were then collected, processed, and imported to the trained SVM and CNN models for prediction. The results showed that all pristine microplastics were correctly identified by both models, of which particles 1–6 were 100% identified by both models. The SVM model yielded a 100% accuracy for particle 7 whereas the CNN model predicted the particle as 99.12% PE, 0.87% PS, and 0.01% nylon. While there is a 0.88% probability that the sample was predicted as other types, this minor discrepancy is unlikely to pose a significant issue for regular applications involving marine microplastics.

To further validate the identification task, we examined 19 randomly selected microparticles from different size ranges across the channel, as illustrated in Fig. 4b. Note that although PS particles were not fluorescent, they could still be distinguished from PE particles under the microscope due to the noticeable difference in size. The results showed that most of the PS particles were captured in zone C (trapping size 10-6 µm) with only particle 1 (PS-1, 10 µm) being captured in Zone A next to a large PE particle. All 19 particles were 100% identified as the correct microplastic. Taken together, our model exhibited promising performance in the identification of small-sized pristine microplastics. Nevertheless, it is important to acknowledge the need for further validation and testing in diverse experimental settings to establish its robustness and generalizability.

### Identification of trapped particles from seawater

**Figure 5:**
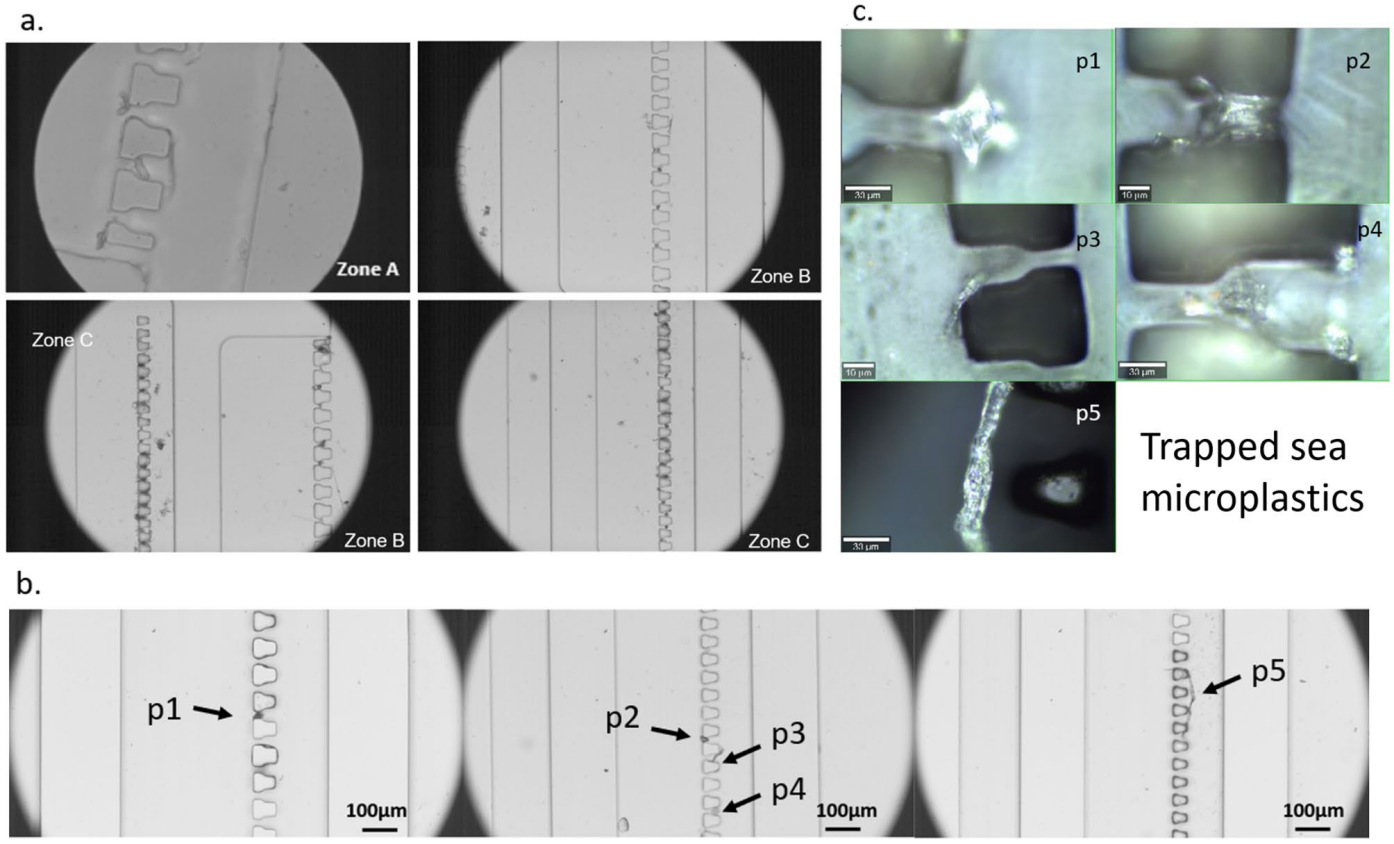
Images that illustrate the trapped particles from the seawater. (**a**) The trapped microparticles were collected from the sea surface water; (**b**) The trapped microparticles after the samples were treated by 30% (v/v) H_2_O_2_; (**c**) particles are observed under a Raman microscope using 50× and 100× objectives. Particles 1, 2, and 4 are in bead forms. Particles 3 and 5 are in fiber forms.

Lastly, the proposed approach for microplastic trapping and identification was further evaluated using environmental samples. A bucket of seawater sample was collected from a local beach and particles were subsequently trapped in the device and transported back to the lab for Raman spectra acquisition. The trapped microparticles collected from on-site sampling are shown in Fig. 5a. Given that particles with associated organic matter and coated biofilms were not the primary focus of this paper and will be addressed in future studies, a sample processing method based on 30% (v/v) hydrogen peroxide (H_2_O_2_) from previous studies was applied^33^. Figure 5b shows the trapped microparticles after the process. The findings suggest that the initial obstruction caused by the dirt in zones B and C was successfully eliminated through peroxide oxidation. Figure 5c presents the particles to be identified under the Raman microscope. We used both CNN and SVM models to identify the trapped microparticles based on the raw Raman spectra (Fig. S2). The identification results were validated with KnowItAll (John Wiley & Sons, Inc, Hoboken, NJ), a widely utilized Raman spectra identification software that employs the reference spectra matching method, and Open Specy, an online community and accessible tool Raman and IR spectra identification. Table 3 shows the identification results, along with the top three prediction outcomes obtained from KnowItAll and Open Specy^34^.

**Table 3:** Prediction results for the five trapped particles from seawater.

Particle number	SVM	CNN	KnowItAll	Open specy
P1	PE	100% PE	52.79% 2-(Ethylsulfonyl) ethanol; 52.55% p-(Ethylene); 51.25% Phosgenite	0.74-Polyamid 6; 0.7-Low density polyethylene; 0.69-polyethylene resin
P2	PE	100% PE	63.37% Thallium(I) chloride; 63.24% Ammonium hexachloroplumbate; 57.22% MS 455;	0.7-polycarprolactone; 0.64-Hydroxyethyl cellulose; 0.61-ethyl cellulose
P3	CA	100%CA	71.3% Zinc Oxide; 70.47% Cellulose, microcrystalline; 69.63% Cellulose;	0.75-Braunite; 0.71-Elbaite; 0.7-Cerulblu
P4	PE	72.42%PE 27.58% PS	62.56% Poly (ethylene-co-vinyl acetate),14 wt. % vinyl acetate; 61.14% p-(Acrylic acid); 60.01% MS 455	0.79-polyamid; 0.75 Low density polyethylene; 0.75-Poly(vinyl-stearate)
P5	CA	69.88% Polyester; 23.67% CA; 6.45% PP	71.96% Cellulose, microcrystalline; 67.52% Sodium borohydride on aluminum oxide; 66.73% Cellulose Acetate sorbate	0.8-Afmite; 0.77-Braunite; 0.73-Arsenopyrite

Overall, the prediction results by our machine learning models for particles were compared with two popular Raman spectrum identification software, KnowItAll, and Open Specy^34^. Particles 1 and 4 were consistently identified as polyethylene by all three methods. To validate the identification result of particle 2, the raw Raman spectra were visually analyzed, specifically by matching the fingerprint peaks for PE^35^. The misclassification by KnowItAll might be caused by the low signal-to-noise (SNR) compared with the other particles. In addition, although Open Specy has a larger spectrum library, it does suffer from some overlapping peaks that match with other materials instead of polymers. It is worth mentioning that due to the different identification results obtained from the three resources, it is recommended to conduct cross-validation using additional Raman spectrum identification tools in the future. This validation process could be further enhanced by coupling it with other analysis tools such as FTIR and imaging analysis. The identification results for particles 3 and 5 are similar between machine learning method and KnowItAll, but they differ from the results obtained with Open Specy. This discrepancy can be attributed to the larger spectrum library and lower data quality of Open Specy. Consequently, there is a possibility that particles 3 and 5 may not be polymers, and the machine learning algorithm could have provided false positive predictions.

When it comes to the Raman identification of sub-micron environmental microplastics, traditional peak matching method often benefits from a cleaner Raman spectrum, which can be achieved through the use of high Raman laser power. However, it is important to note that employing high Raman laser power carries the risk of potentially burning the particles. Moreover, these conventional methods often involve additional processing steps that can result in damage to the plastics, compromising the integrity of the Raman spectra. With the advantages given by machine learning technologies, complex variations in the spectra originating from various environmental conditions may be correctly interpreted, future focus should focus on how different additives, weathering degradation, along with biofouling can affect the spectra and if machine learning models can help us unravel the underlying mystery fingerprints.

To improve the separation and trapping ability of the device, a combination with a high throughput separator and other active particle trapping techniques could be used, such as a hydrocyclone^36^. Moreover, particle recycling from the microfluidic system should also be considered to further improve the system for other downstream studies. One possible solution is to add microelectrodes that generate negative dielectrophoresis to selectively release target particles^37^. Overall, the device is beneficial for accurate microplastic detection, primarily due to its capability to trap an extremely small number of particles in a single trap. This feature simplifies the task of focusing on individual particles during Raman analysis, which can be challenging when using conventional methods like filter papers where particles can be embedded in deeper fiber layers^38^. Another main advantage of using the microfluidic device over the conventional sampling methods is the effective reduction of cross-contamination from atmospheric particles, as the microplastics remain trapped within the channel throughout the entire process without exposure to the surrounding atmosphere.

In summary, this paper introduces a promising microfluidic device specifically designed for efficient microplastic trapping and identification. The proposed trapping method holds great potential for minimizing cross-contamination and decreasing the reliance on manual labor. It demonstrates efficient trapping of pristine microplastic particles, even in small quantities, while also offering size-selective capabilities. Experimental tests have successfully demonstrated the device's capability to effectively trap environmental microplastic particles in real seawater. These positive results provide encouraging evidence of the device's practicality and effectiveness in real-world conditions. However, it is important to acknowledge that there are still concerns that need to be addressed to further improve its performance. For instance, one way to enhance the scalability of this system is by implementing parallelization and connecting multiple devices in series. This approach can increase the overall throughput and efficiency of the trapping process. Additionally, while the current particle trapping method relies on hydrodynamic force at a low flow rate, integrating other active-driven methods with the chip has the potential to further boost the throughput. Moreover, the integration of hand-held Raman spectroscopy with advanced machine-learning identification systems holds great potential for continuous on-site monitoring of microplastics^39^.

To facilitate the adoption of this system for long-term environmental monitoring, cost-effectiveness becomes a crucial consideration. One viable approach to mitigating the cost of fabricating microfluidic devices is directly using 3D printing technology to create the devices themselves. This approach eliminates the need for molds and streamlines the manufacturing process, thereby reducing costs^40^. However, it is important to pay attention to the materials used for 3D printing the microfluidic devices. Given the focus on microplastic analysis, it is essential to consider the potential risks of introducing additional plastic particles during the manufacturing process. Careful selection of appropriate 3D printing materials that minimize the release of microplastics is necessary to ensure the integrity of the analysis and avoid introducing unwanted contaminants.

Furthermore, due to variations in shape, size, and unique characteristics of environmental particles, it is crucial to consider these factors for optimal performance. However, capturing and identifying environmental nanoplastics present significant challenges. Integrating this system with other active-driven methods can provide a more effective approach, particularly for nanoplastics. By combining the capabilities of this microfluidic device with active-driven methods designed for nanoplastic analysis, a comprehensive characterization of nanoplastics in environmental samples could be achieved in future studies^41–43^.

Additionally, expanding the Raman spectrum data library to include a wider range of weathered polymers and incorporating Raman spectra of other materials in the training dataset is essential. This expansion will enhance the capability of machine learning models to identify various types of environmental particles and reduce the false positive rate^44^. It also provides an opportunity to assess the strengths and weaknesses of different machine learning algorithms, enabling a comprehensive analysis and selection of the most suitable models for accurate and reliable predictions. Moreover, through the utilization of data segmentation techniques and conducting in-depth imaging analyses, we can expect to gain a deeper understanding of valuable insights concerning the particles and their interconnected environmental contexts^45,46^. Overall, the knowledge would contribute to a more comprehensive understanding of the sources, fate, and transport of environmental particles, enabling targeted interventions and mitigation strategies to be implemented.

## Materials and methods

### Plastic samples

A total of 11 types of plastics representing commonly collected marine microplastic pollutants were studied, including polystyrene (PS), polypropylene (PP), polyethylene (PE), polyamide (PA, Nylon), polyester, polyethylene terephthalate (PET), polyvinyl chloride (PVC), polyurethane (PUR), polycarbonate (PC), Poly(methyl methacrylate) (PMMA), and cellulose acetate (CA). The PC and PUR samples were prepared from clear sheets and tubing, respectively. The rest of the samples were prepared using the polymers in Polymer Kit 1.0 (Hawai’i Pacific University Center for Marine Debris Research). They came in various forms including pellets, fibers, beads, and powder. Particles used in the microfluidic trapping experiments are PE (20–27 µm and 10–45 µm) and PS (9.5–11.5 µm) microspheres from Cospheric LLC. (Goleta, California, USA). To construct the machine learning testing dataset, more samples of common daily plastic products were also applied (Table S1).

### Data acquisition

#### Confocal Raman spectroscopy

A WITec alpha 300 R confocal Raman microscope (CRM) was used in this study. It is equipped with two excitation laser wavelengths, 532 and 785 nm. The diffraction gratings used were 1200 g/mm and 300 g/mm for 532 nm and 785 nm, respectively. Different magnification power and objectives (10X, 50X, and 100X) were used based on the sample sizes to obtain the most comprehensible Raman spectra. All spectra were collected with an accumulation time of 1 s and 100 iterations. A full range of wavelength shifts (10–4000 cm^−1^) was collected for all samples and was later truncated to 300–2000 cm^−1^ representing the fingerprint region, which is generally sufficient for material identification^47^.

#### Data acquisition for large-sized plastic samples

The pristine plastic samples from the polymer kit were tested on a regular microscope glass slide, most of the samples are 3–5 mm pellets. The CA sample comes in the form of powder, the average particle size (longest dimension) is approximately 0.387 mm. The polyester sample is a white fabric and was cut into 5 by 5 mm squares for testing. 10 samples were prepared for each type of plastic, among which five samples were tested with a 532 nm excitation wavelength whereas the other five samples were tested with a 785 nm wavelength. All data were collected with a 10X objective, and three Raman spectra were collected for each sample with different laser powers, 5 mW, 10 mW, and 15 mW. Thus, a total of 10 × 3 (5, 10, 15 mW) = 30 raw spectra were collected for each type of pristine plastic. Daily plastic products were collected and tested once for each sample with the most appropriate magnification and laser power. Note that clear samples such as the PC clear sheets were tested using 532 nm wavelength thanks to their negligible fluorescence background, while color dyed samples such as black polyester threads were tested with 785 nm wavelength because higher excitation wavelength can decrease fluorescence background and provide a better signal-to-noise ratio^48^. In sum, a total of 330 data points were collected from pristine materials, and 59 data points from daily products.

#### Data acquisition for trapped microplastics in the microfluidic device

Mixed samples of PE and PS microspheres were injected into the microchannel and trapped by the sieve-like structures to prove the concept of in situ microplastic trapping and identification in microfluidic devices. Note that though the microfluidic channel was made of PDMS by standard soft lithography, a glass slide was placed on top of the channel to form an enclosed channel without permanent bonding. As such, the cover can be removed if needed, to eliminate potential background signals on smaller particles. Trapped particles were tested using 50× and 100× objectives under 785 nm excitation wavelength and various laser intensities depending on their sizes to collect Raman spectra.

### Dataset construction

To develop a comprehensive training dataset, two open-source microplastic Raman data repositories, spectral libraries of regular plastics (SLOPP) and environmental weathered database (SLOPPE)^49^, and a Mendeley database containing spectra collected from both standard and naturally weathered samples^50^, were adopted to complement the raw data collected from pristine samples described above. Taking into account the quantity and quality of the data, we made the decision to incorporate all the data from the SLOPP library and a portion of the Mendeley library into the training dataset. This choice was made because some spectra in the Mendeley dataset originated from severely weathered samples, which rendered them unable to provide accurate ground truth information. We further added the data from SLOPPE library and 10 extra pristine data points per plastic type to the testing dataset. Eventually, the original training dataset contains 587 data points (Table S2), and the testing dataset contains 265 data points (Table S3).

For further data processing, we modified Raman spectra in training and testing datasets from all sources to the desired Raman fingerprint wavenumber range of 300–2000 cm^−1^ and adjusted the data to have the same input dimension by using WebPlotDigitizer^51^. Moreover, several data augmentation techniques were implemented to expand the training datasets. First, additive white Gaussian noise (AGWN) was added to all the original data points, mimicking the generic spectral noises (e.g., shot noise, dark noise, and readout noise) generated naturally^52,53^. Five SNRs were applied in the data augmentation process with overall shapes and peaks of the spectra retained. Lastly, the augmented data were merged with the original spectra datasets. Subsequently, the polynomial baseline removal technique was applied to all augmented data sourced from both the SLOPP and Mendeley libraries. This step was crucial in mitigating the influence of noisy background signals present in these spectra. Specifically, we used the Polynomial Features function from the preprocessing library of scikit-learn^54^, and picked three appropriate exponent values (degrees) for each data point. Herein, the number of data points from SLOPP and Mendeley libraries was tripled from the previous augmentation step. Adding all together, a total of 11,772 training data points were obtained (Table S4). It is worth noting that the test dataset was not subjected to augmentation. Finally, we standardized all data points, specifically the Raman intensity, to a range between 0 and 1. A visual representation of the data processing steps for a weathered PP sample can be observed in Fig. S2.

### Machine learning models

**Figure 6:**
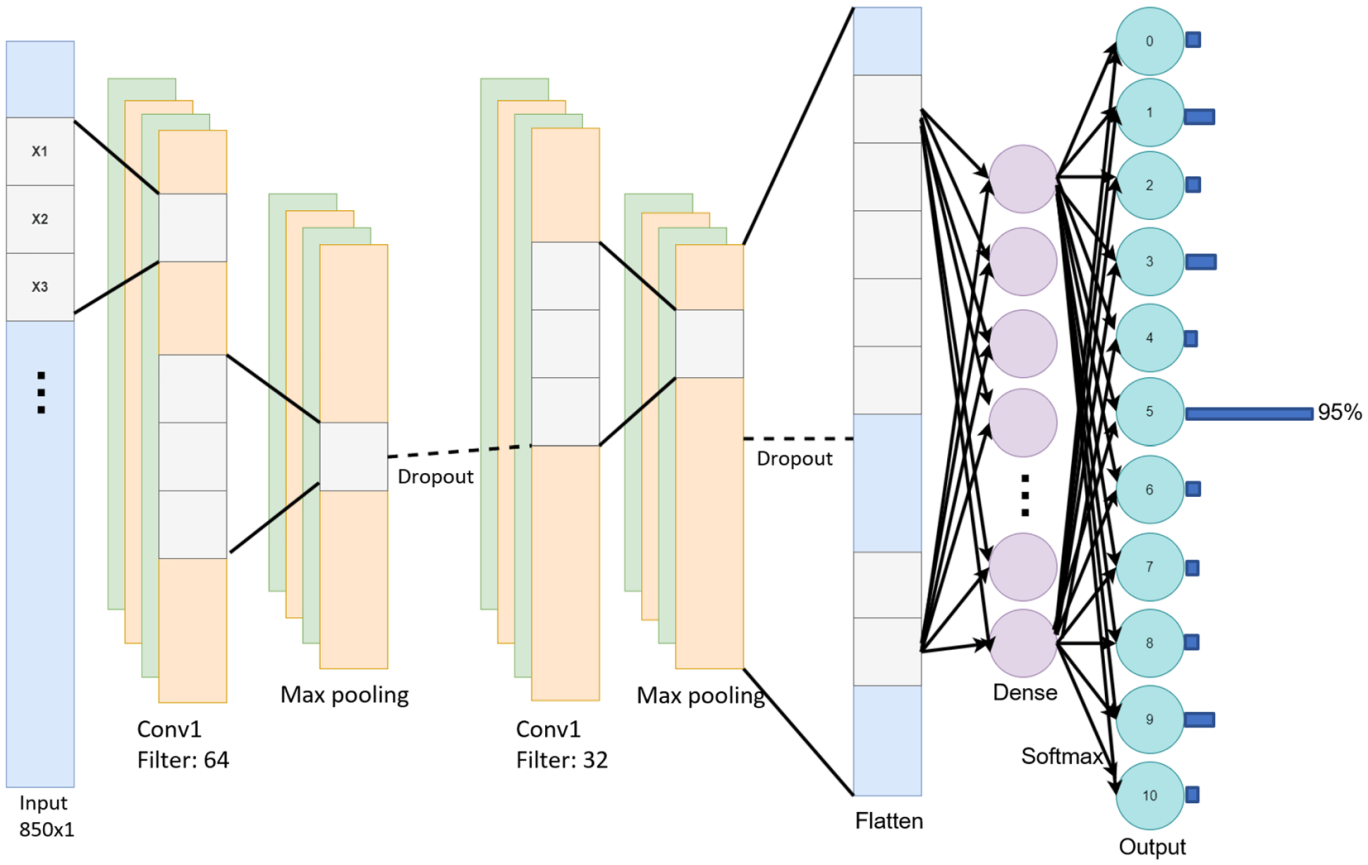
CNN architecture used in this paper. The architecture contains two convolution layers. The first convolution layer has a filter of size 64. The second convolution layer has 32 filters.

Classification or identification tasks based on Raman spectroscopy and machine learning have been extensively explored in previous studies. These investigations have demonstrated the feasibility of this approach and have reported promising results^55–70^. CNN and ResNet have emerged as popular choices for such applications, consistently showcasing superior identification accuracy compared to other models. The microplastic identification application reported by Ramanna et al. 2022^71^ capitalized on an RF model and trained the model using the database SLOPP, followed by the tests on the database SLOPPE and achieved 93.81% accuracy. In this paper, we implemented four machine learning models (i.e., SVM, RF, CNN, and ResNet34 models) and comprehensively compared their performances in identifying microplastics from various sources. We used the SVM and RF libraries from scikit-learn. We constructed a CNN model using the Conv1D feature from the Keras library (Fig. 6). The CNN model starts with an initial 1D input layer that contains a total of 850 data points. The inner layers of the models comprise two convolutional layers, each followed by a max pooling layer and a drop-out layer with an initial drop-out rate of 0.3, and a fully connected layer applied after. A softmax activation function was applied in the output layer for final prediction. Here, we applied the generic ResNet34 architecture reported in reference^72^ for our task and reshaped the input to a one-dimensional vector. Hyperparameters used were determined based on Grid Search and the tenfold cross-validation methods (Table S5).

### Evaluation metrics of model performance

Classification accuracy is a crucial metric that determines the performance of an algorithm on the performance of microplastic identification. A straightforward method to visualize and interpret this metric is to plot the training and validation accuracy, and the history of cross-entropy loss. However, this metric alone is insufficient to evaluate the robustness of the prediction accuracy of the model, especially for imbalanced datasets^73^. To address this issue, several evaluation metrics that are well known for multiclass classification applications, such as confusion matrix, F1 score, and MCC score were applied. In each training fold, the data points are split into 80% training data and 20% validation data. The average classification accuracy was obtained after 10 folds. A confusion matrix is visualized to evaluate the classification accuracy of the model on the held-out test dataset. We also adopted matrices including macro-average F1 score, weighted-average F1 score, and Matthews correlation coefficient (MCC) to evaluate the overall classification performance for all models.

We plotted the ROC curve (receiver operating characteristic curve) and compared the AUC (area under the ROC curve) using One vs Rest (OvR) method to evaluate the prediction precision for a particular class to understand how well the models distinguish between classes. The ROC AUC evaluates the classification capability of the models at various thresholds. In this case, we want the threshold to be the highest TPR and lowest FPR. We calculated the optimal threshold by finding the point that maximizes TPR-FPR on the micro-average ROC curve, which is represented by Youden's index, $J$^74^:

1 $$J=sensitivity+specificity-1$$

Sensitivity is the y-axis of the ROC curve, which is calculated as

2 $$sensitivity= \frac{True \, positives}{True \, positives+False \, negatives}$$

The x-axis of the ROC curve represents 1-specificity, which is calculated as

3 $$1-specificity= \frac{True\,negatives}{True \, negatives+False \, negatives}$$

### Microplastic trapping and identification in microfluidic devices

As stated above, previous studies have long overlooked the microplastics at micro- and submicron scales, which in fact are one of the most abundant and concerning pollutants in seawater. Leveraging the advantages of the particle trapping ability of microfluidics, a passive hydrodynamic trapping device based on predesigned microstructures or trapping cages was adopted^75^, which does not require external driving fields and is user-friendly for laypersons.

**Figure 7:**
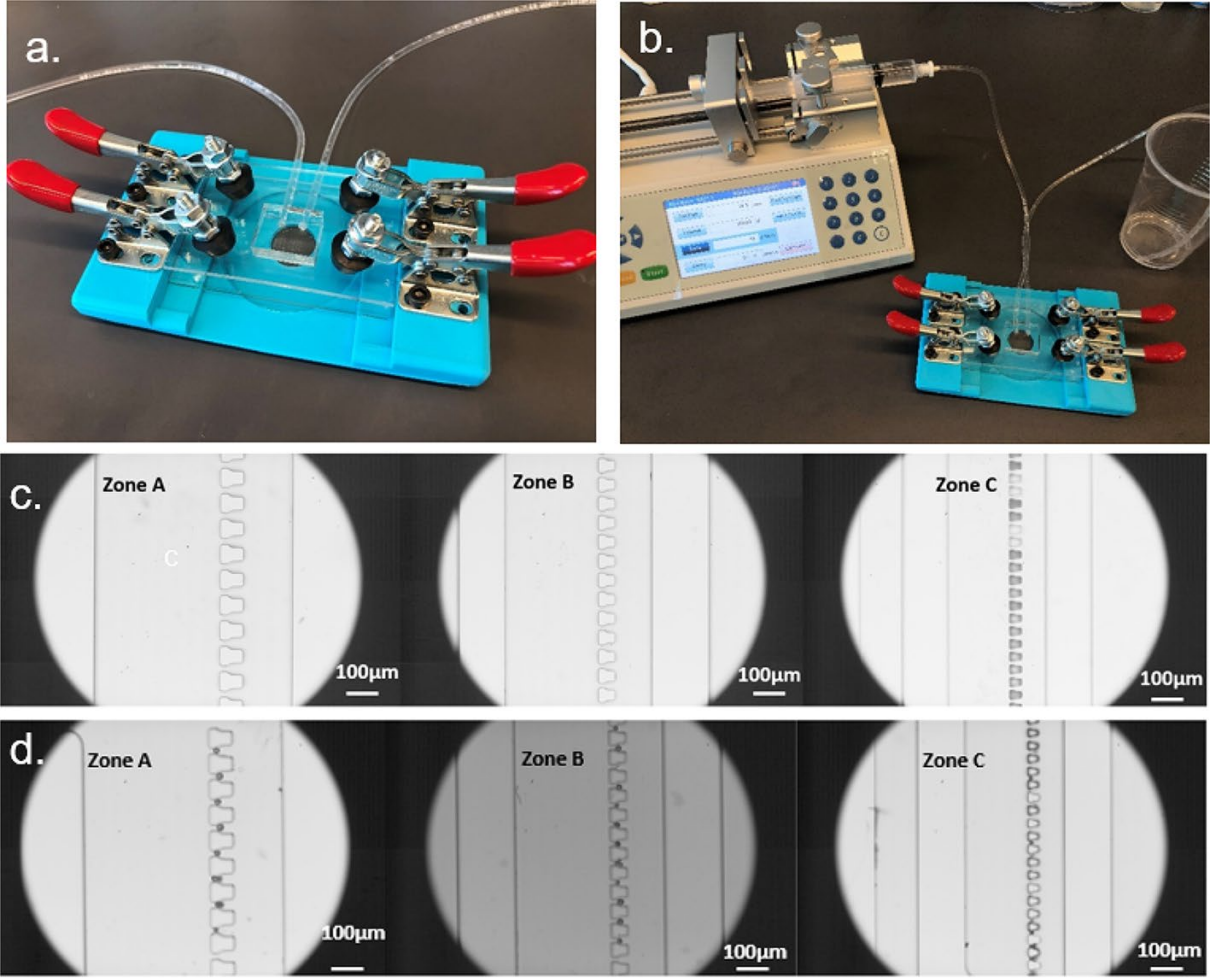
Configuration of the microfluidic device and proof of concept experiment set up. (**a**) The microfluidic device assembled on the holding fixture; (**b**) Photo illustrating the experimental setup for in-situ testing. The setup includes a syringe pump for supplying inlet flow at a constant flow rate, and a collection cup for receiving the outlet waste; (**c**) The three trapping zones of different target particle sizes; (**d**) The particles are trapped and separated in small amounts in sieve-like structures of corresponding sizes.

The microfluidic device developed in this paper is similar to traditional PDMS-glass devices made from standard soft lithography whereas permanent bonding was not carried out. To avoid leakage, a 3D printed fixture was applied as shown in Fig. 7a. To inject the microplastic samples into the device (Fig. 7b), a syringe pump (Fusion 200, Chemyx Inc., Stafford, TX, USA) was used, and wastes were collected in a disposable cup. Note that, the trapping occurred in three different-sized sieve-like traps (Fig. 7c) that will be used to trap microplastics with corresponding size ranges, 45–21 µm (zone A), 20–11 µm (zone B), and 10–6 µm (zone C). All other dimensions of the channel were maintained in accordance with the recommended ratios specified in the reference^75^.

### Proof-of-concept particle trapping and identification

The proof-of-concept experiment was initially done in the lab by flowing mixed PS (9–11.5 µm), non-fluorescent PE (10–45 µm), and fluorescent PE (20–27 µm) particles suspended in deionized (DI) water into the channel. Note that the fluorescence was only used to differentiate microplastics visually, which provides an alternative approach to recognizing microplastics for validation purposes. To do so, 10 mg of particle samples were mixed in 200 mL distilled water and injected into the device with a flow rate of 10 µl/min. Microspheres were successfully captured in corresponding-sized sieve-like traps as shown in Fig. 7d. Afterward, the microfluidic device was gently removed from the holding fixture and used for Raman analysis.

### On-site sampling and particle identification

**Figure 8:**
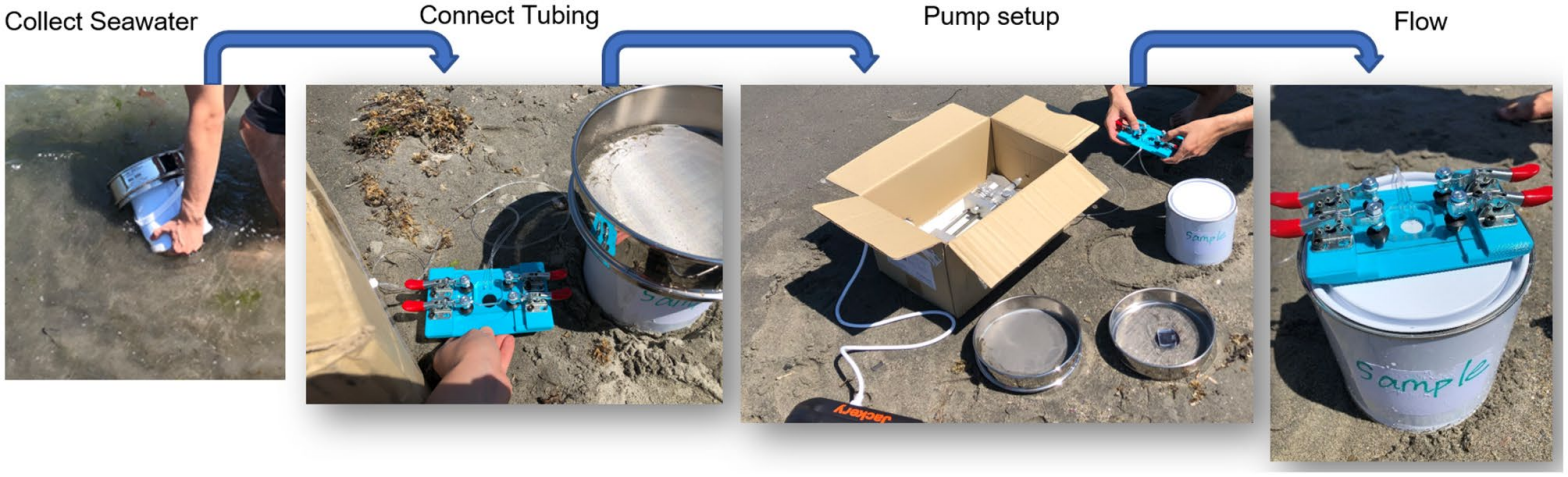
On-site sampling experiment set up. Seawater was first filtered and collected. The syringe pump was powered by a camping battery bank and set to withdrawal mode at a 10 µl/min flow rate. The experiment lasted three hours.

In addition to the indoor tests, onsite trapping of microplastics was also conducted on a local beach to demonstrate the feasibility. Surface seawater was first filtered with 1 mm and 45 µm sieves. The collected seawater sample was sealed inside a thoroughly cleaned stainless-steel bucket. The microfluidic device was connected to the inlet tubing, and the outlet was connected to an empty 10 ml syringe. The syringe pump was configured in withdrawal mode to extract seawater from the bucket. Subsequently, the device was transported back to the laboratory for subsequent analysis. The experiment setup at the beach is shown in Fig. 8.

## Conclusions

In this paper, we combined Raman spectroscopy, machine learning, and microfluidics to develop a novel microfluidic device that traps microplastics down to several microns and systematically examined the performance of several machine learning models (i.e., SVM, RF, CNN, and ResNet34) on microplastic identification. The trained CNN and SVM can identify pristine microplastic particles with near 100% accuracy. Furthermore, the models can identify environmental microplastic particles separated from seawater with high accuracy as well. The size-selective trapping capability of the device greatly benefits more accurate microplastic detection in Raman analysis. In summary, the proposed process holds significant potential for long-term, label-free continuous monitoring and assessment of microplastics in seawater. Moreover, this concept can be readily adapted for analyzing other types of environmental microparticles. Future research endeavors should concentrate on expanding the dataset continuously by incorporating a broader range of environmental samples. Additionally, refining the deep learning models to enhance accuracy and robustness is crucial. For severely degraded microplastics, a cross-validation of identification results and the integration of multiple characterization methods, such as mass spectrometry and energy dispersive spectroscopy, could be considered. Furthermore, parallelization of the device and exploration of alternative separation techniques can enhance throughput and improve the recovery of trapped particles for downstream studies.

## Supplementary Information

Supplementary Information.

## Data Availability

All relevant data is available upon request from the corresponding author Yang Lin at yanglin@uri.edu.

## Code availability

All relevant code is available upon request from the corresponding author Yang Lin at yanglin@uri.edu.
