# Supplementary material for: A microfluidic approach for label-free identification of small-sized microplastics in seawater
Source: Sci Rep. 2023 Jul 7;13:11011. doi: 10.1038/s41598-023-37900-9 (PMC10329028; doi:10.1038/s41598-023-37900-9)
Supplement: Supplementary file 1 — Supplementary Information. [file 41598_2023_37900_MOESM1_ESM.docx]

**Supplementary Material**

**A Microfluidic Approach for Label-Free Identification of Small-Sized Microplastics in Seawater**

Liyuan Gong^1^, Omar Martinez^1^, Pedro Mesquita^1^, Kayla Kurtz^2^, Yang Xu^3^ & Yang Lin^1^*

^1^ Department of Mechanical, Industrial and Systems Engineering, University of Rhode Island, Kingston, Rhode Island, USA

^2^ Department of Civil and Environmental Engineering, University of Rhode Island, Kingston, Rhode Island, USA

^3^Department of Computer Science, San Diego State University, San Diego, California, USA

^*^ **Corresponding author.**

*E-mail address:* [yanglin@uri.edu](mailto:yanglin@uri.edu) (Y. Lin).


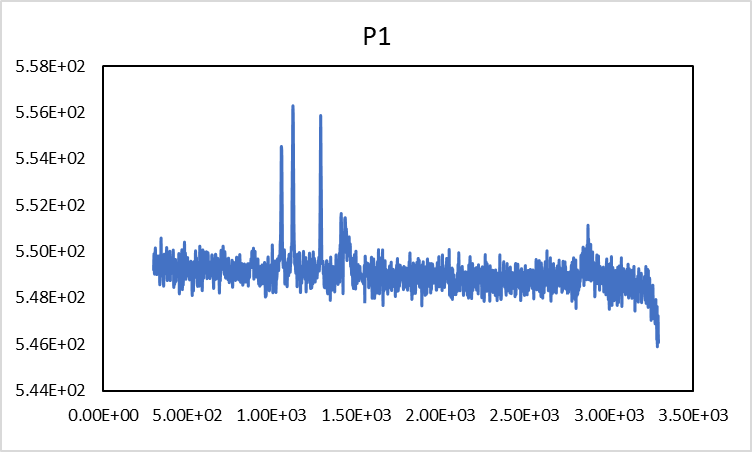

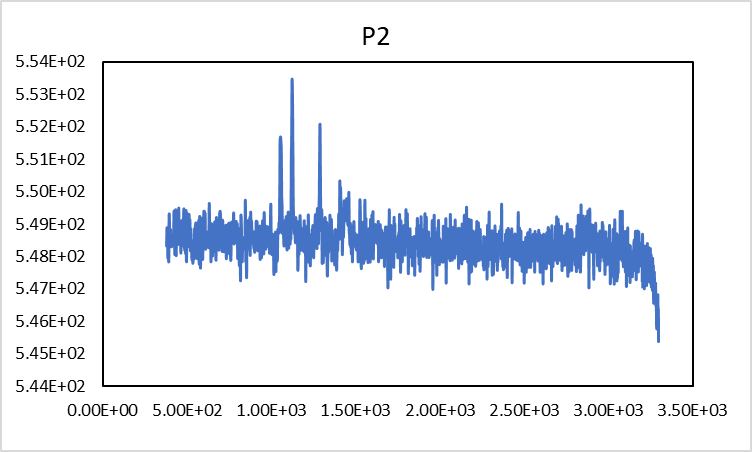


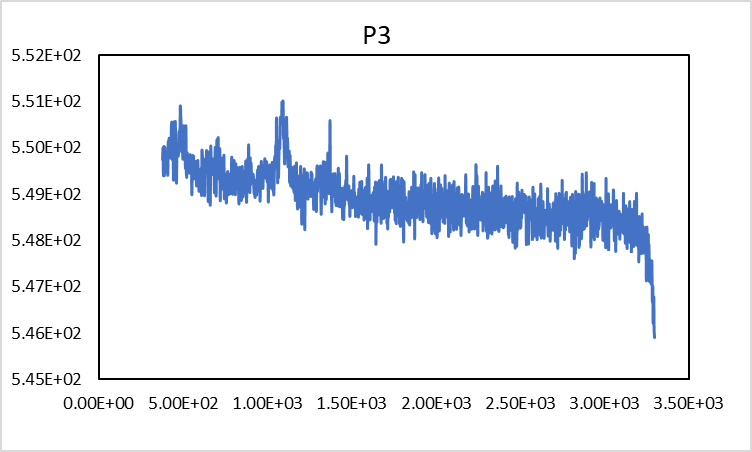

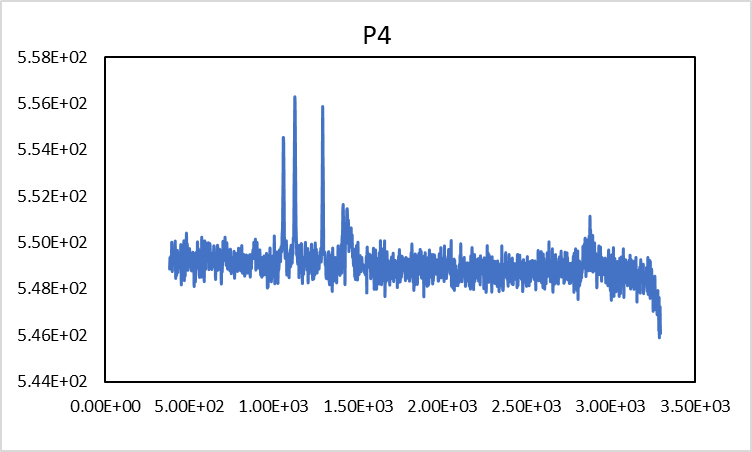


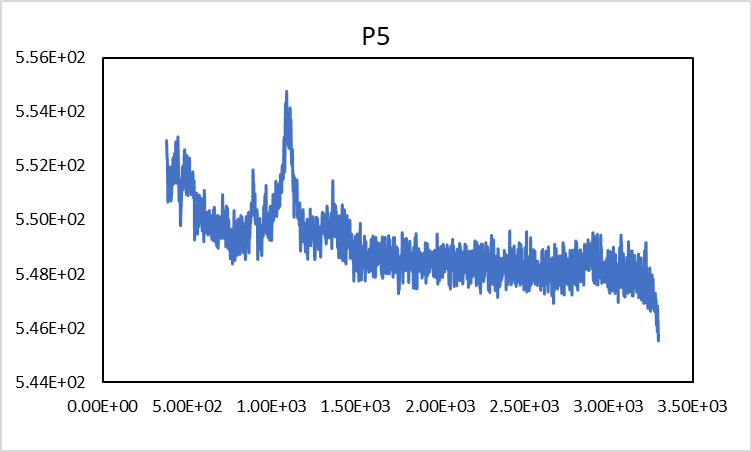


**Fig. S1.** Raw Raman spectra of the five trapped particles from seawater.

**Table S1.** Samples of daily plastic products

| **Plastic-type** | **Product (Qty)** |
| --- | --- |
| PS | Red cup (1), clear bags (3), white cup (1), black and transparent takeout containers (2) |
| PP | PP cotton (1), toy (2), pipette tip (1), transparent container (2), Multicolor microcentrifuge Tubes (4) |
| PET | Water bottle (1), hand soap bottle (1), sprite bottle (1), strawberry container (1), bottle containers (2) |
| Polyester | Clothing (3), multicolor threads (5) |
| PA, Nylon | Fishline (1), syringe connecter (1), black zip tie (1), nut (1), 3D printing resin (1), white thread (1) |
| PC | Safety glasses (2), clear sheet (1), pitcher (1) |
| PE | Multicolor Shampoo bottle (3), transparent bag (2), |
| PU | Air hose tubing (1), mattress foam (1) |
| PVC | Fitting (1), garbage can (1), white sheet (1), tape (1) |
| PMMA | Bank card (1), ID card (1), clear PMMA sheet (2), acrylic nails (1) |
| CA | Cigarette filter (1), black hair comb (1) |

**Table S2.** Original training dataset (before augmentation, 587 data points)

| **Plastic type** | **Pristine plastic** | **SLOPP** | **Mendeley** | **Total** |
| --- | --- | --- | --- | --- |
| PS | 30 | 11 | 1 | 42 |
| PP | 30 | 17 | 51 | 98 |
| PET | 30 | 9 | 0 | 39 |
| Polyester | 30 | 10 | 16 | 56 |
| PA, Nylon | 30 | 7 | 4 | 41 |
| PC | 30 | 7 | 2 | 39 |
| PE | 30 | 24 | 68 | 122 |
| PU | 30 | 6 | 0 | 36 |
| PVC | 30 | 11 | 8 | 49 |
| PMMA | 30 | 1 | 0 | 31 |
| CA | 30 | 4 | 0 | 34 |

**Table S3.** Testing dataset

| **Plastic type** | **Pristine plastic** | **Daily product** | **SLOPPE** | **Total** |
| --- | --- | --- | --- | --- |
| PS | 10 | 7 | 9 | 26 |
| PP | 10 | 10 | 21 | 41 |
| PET | 10 | 6 | 1 | 17 |
| Polyester | 10 | 8 | 12 | 30 |
| PA, Nylon | 10 | 6 | 6 | 22 |
| PC | 10 | 4 | 2 | 16 |
| PE | 10 | 5 | 26 | 41 |
| PU | 10 | 2 | 6 | 18 |
| PVC | 10 | 4 | 3 | 17 |
| PMMA | 10 | 5 | 3 | 18 |
| CA | 10 | 2 | 7 | 19 |

**Table S4.** Final training dataset (after augmentation, 11,772 data points)

| **Material Type** | **Pristine plastic** | **SLOPP** | **Mendeley** | **Total** |
| --- | --- | --- | --- | --- |
| PS | 180 | 396 | 36 | 1152 |
| PP | 180 | 612 | 1836 | 2628 |
| PET | 180 | 324 | 0 | 504 |
| Polyester | 180 | 360 | 576 | 1116 |
| PA, Nylon | 180 | 252 | 144 | 576 |
| PC | 180 | 252 | 72 | 504 |
| PE | 180 | 864 | 2448 | 3492 |
| PU | 180 | 216 | 0 | 396 |
| PVC | 180 | 396 | 288 | 864 |
| PMMA | 180 | 36 | 0 | 216 |
| CA | 180 | 144 | 0 | 324 |


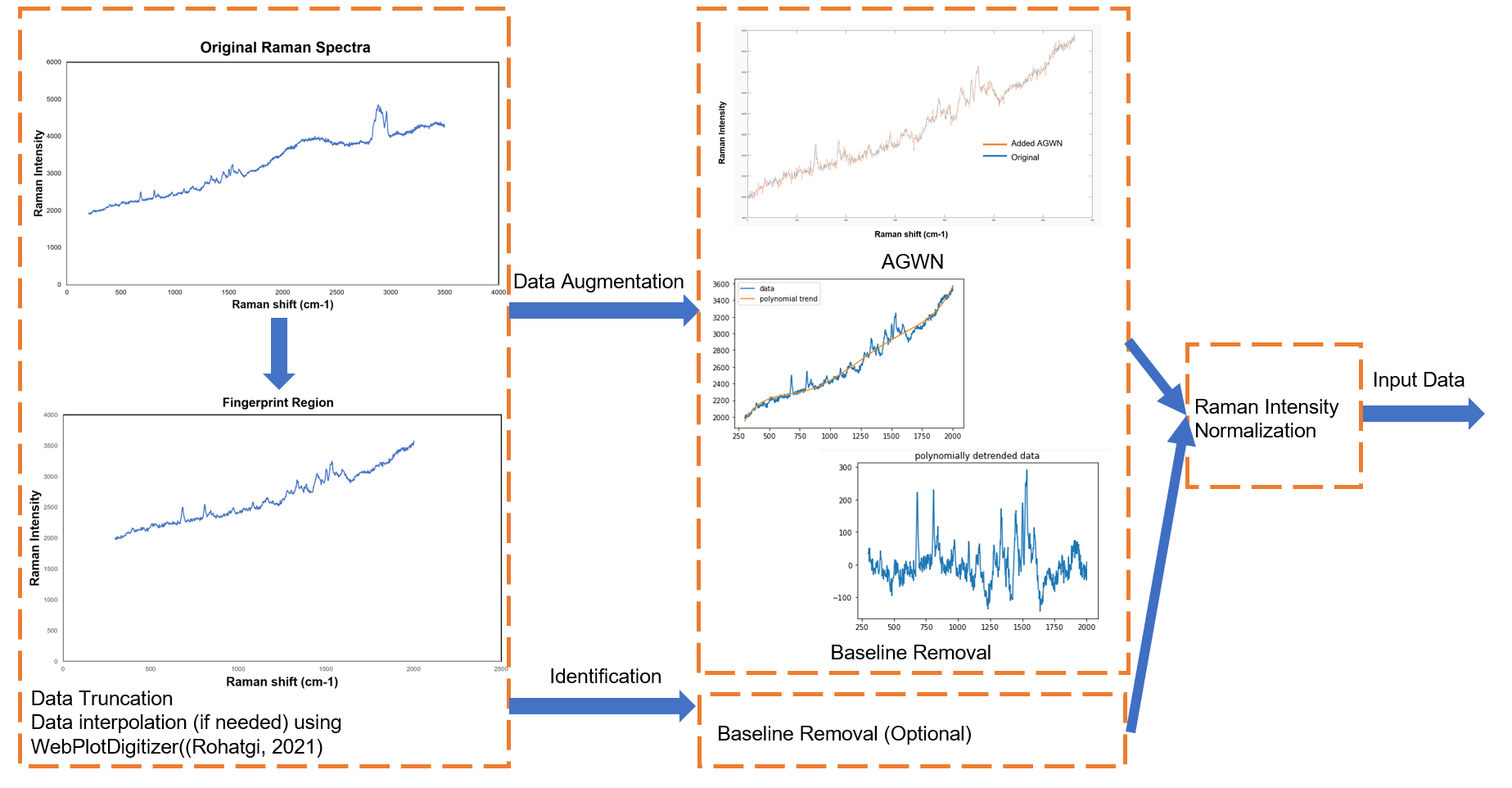


**Fig. S2.** Weathered PP from Mendeley dataset goes through data processing steps in the paper


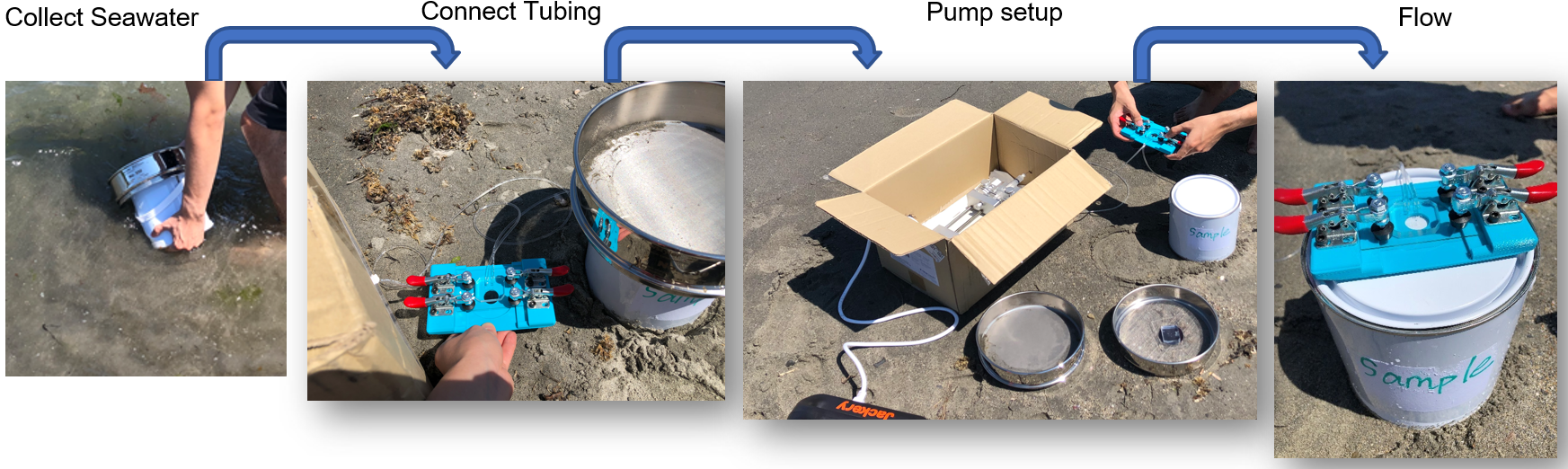


**Fig. S3.** On-site sampling experiment setup at the beach

**Table S5.** Tuned hyperparameters for each model

| Training Dataset | SVM | RF | CNN | ResNet34 |
| --- | --- | --- | --- | --- |
| Original | Kernel function: Linear  Penalty term C=10  No gamma value is needed | N_estimator (number of trees): 100 | Filter size: conv1-64, conv2-256  Batch size: 32  Learning rate: 0.001  Optimizer: Adams  Drop-out rate: 0.5  Epochs: 100 | Batch size: 16  Learning rate: 0.001  Batch normalization momentum: 0.9  Epochs: 120 |
| Augmented | Kernel function: Linear  Penalty term C=10  No gamma value is needed | N_estimator (number of trees): 100 | Filter size: conv1-64, conv2-256  Batch size: 64  Learning rate: 0.001  Optimizer: Adams  Drop-out rate: 0.5  Epochs: 80 | Batch size: 64  Learning rate: 0.001  Batch normalization momentum: 0.9  Epochs: 120 |


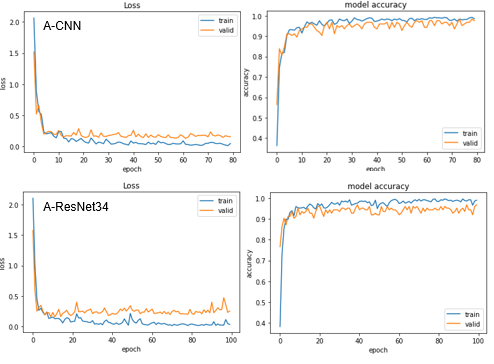


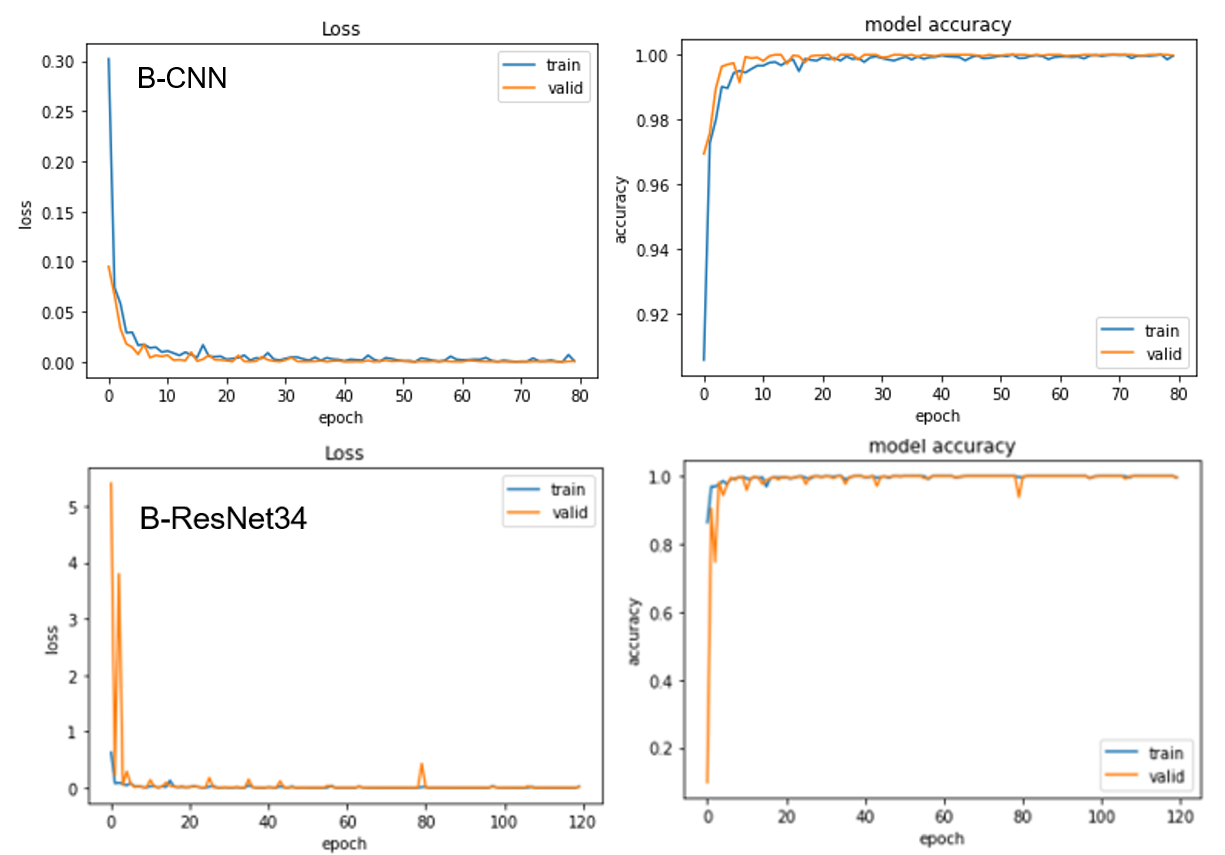


**Fig. S4**. Training accuracy, Validation accuracy, and Cross Entropy loss history of CNN and ResNet34. A. trained with the original dataset; B. trained with the augmented dataset.

**Reference**

Rohatgi, A. (2021). *WebPlotDigitizer* (4.5).
